# Risk of sexually transmitted infections among U.S. military service members in the setting of HIV pre-exposure prophylaxis use

**DOI:** 10.1371/journal.pone.0296054

**Published:** 2023-12-28

**Authors:** Jason M. Blaylock, Evan C. Ewers, Elizabeth J. Bianchi, David B. King, Rosemary O. Casimier, Hector Erazo, Stephen Grieco, Jenny Lay, Sheila A. Peel, Kayvon Modjarrad, Charmagne G. Beckett, Jason F. Okulicz, Paul T. Scott, Shilpa Hakre

**Affiliations:** 1 Infectious Diseases Service, Walter Reed National Military Medical Center, Bethesda, Maryland, United States of America; 2 Infectious Diseases Service, Fort Belvoir Community Hospital, Fort Belvoir, Virginia, United States of America; 3 Henry M. Jackson Foundation for the Advancement of Military Medicine, Inc., Bethesda, Maryland, United States of America; 4 U.S. Military HIV Research Program, Walter Reed Army Institute of Research, Silver Spring, Maryland, United States of America; 5 Emerging Infectious Diseases Branch, Walter Reed Army Institute of Research, Silver Spring, Maryland, United States of America; 6 Level One Personnel, Columbia, Maryland, United States of America; 7 Preventive Medicine Branch, Walter Reed Army Institute of Research, Silver Spring, Maryland, United States of America; 8 Diagnostics and Countermeasures Branch, Walter Reed Army Institute of Research, Silver Spring, Maryland, United States of America; 9 Navy Bloodborne Infection Management Center, Bethesda, Maryland, United States of America; 10 Infectious Diseases Service, San Antonio Military Medical Center, Fort Sam Houston, Texas, United States of America; University of New South Wales, AUSTRALIA

## Abstract

**Background:**

The evidence for an increased incidence of sexually transmitted infections (STIs) among patients utilizing HIV pre-exposure prophylaxis (PrEP) has been inconsistent. We assessed the risk of incident STI while on PrEP compared to periods off PrEP among military service members starting PrEP.

**Methods:**

Incidence rates of chlamydia, gonorrhea, syphilis, hepatitis C virus, and HIV were determined among military service members without HIV prescribed daily oral tenofovir disoproxil fumarate and emtricitabine for HIV PrEP from February 1, 2014 through June 10, 2016. Hazard ratios for incident STIs were calculated using an Anderson-Gill recurrent event proportional hazard regression model.

**Results:**

Among 755 male service members, 477 (63%) were diagnosed with incident STIs (overall incidence 21.4 per 100 person-years). Male service members had a significantly lower risk of any STIs (adjusted hazard ratio (aHR) 0.21, 95% CI 0.11–0.40) while using PrEP compared to periods off PrEP after adjustment for socio-demographic characteristics, reasons for initiating PrEP, surveillance period prior to PrEP initiation, and the effect of PrEP on site and type of infection in multivariate analysis. However, when stratifying for anatomical site and type of infection, the risk of extragenital gonorrhea infection (pharyngeal NG: aHR 1.84, 95% CI 0.82–4.13, p = 0.30; rectal NG: aHR 1.23, 95% CI 0.60–2.51, p = 1.00) and extragenital CT infection (pharyngeal CT: aHR 2.30, 95% CI 0.46–11.46, p = 0.81; rectal CT: aHR 1.36, 95% CI 0.81–2.31, p = 0.66) was greater on PrEP compared to off PrEP although these values did not reach statistical significance.

**Conclusions:**

The data suggest entry into PrEP care reduced the overall risk of STIs following adjustment for anatomical site of STI and treatment. Service members engaged in PrEP services also receive more STI prevention counseling, which might contribute to decreases in STI risk while on PrEP.

## Introduction

Consistent use of HIV preexposure prophylaxis (PrEP) reduces sexual risk of HIV acquisition by 99% among men-who-have-sex-with-men (MSM) [1]. Since its first approval for HIV prevention in 2012 by the United States (U.S.) Food and Drug Administration (FDA), tenofovir disoproxil fumarate/emtricitabine (TDF/FTC) prescriptions among eligible persons increased from 3% in 2015 to 25% in 2020 [2]. In an effort to increase uptake, federal initiatives in 2019 led to PrEP becoming available free of cost to anyone at high risk for HIV infection regardless of income with private or public insurance [2, 3]. Although HIV incidence in the U.S. decreased by 8% from 2015 to 2019, reportable sexually transmitted infections (STIs) such as chlamydia, gonorrhea, and syphilis increased by 30% in the same period [4, 5].

Several studies have noted a rise in sexual behaviors that increase the risk of STIs following PrEP initiation including more sex partners and increased rates of condomless anal sex and sexualized drug use [6–8]. Reported changes in STI incidence rates and prevalence proportions in MSM PrEP populations have been variable. Some studies demonstrated an association between PrEP use and STI rates following PrEP initiation, although these associations are variable [6–12]. In contrast, a few studies have shown stable rates of STIs following PrEP initiation, suggesting that the impact of PrEP on risk behaviors and STI incidence is heterogenous in different populations. Additionally, it is difficult to determine if the increased number of STI diagnoses is consequent to increased frequency of STI testing, as endorsed in the Centers for Disease Control PrEP guidelines [13]. Of particular importance is the role of extragenital infection in MSM, which can account for >50% of chlamydia or gonorrhea infections and is often asymptomatic [14]. Furthermore, a recent prospective study of three site screening by Zucker and colleagues found that MSM on PrEP had the highest rates of extragenital gonorrhea or chlamydia with a prevalence of 47% compared with HIV-infected (20%) and MSM not HIV-infected and on PrEP (22%) [15]. Importantly, 98% of the infectious would have been missed with urogenital screening alone.

The Department of Defense (DoD) Military Health System (MHS) provides health services to 9.6 million beneficiaries. Early adoption of HIV PrEP services within the MHS was driven by infectious diseases subspecialty services located at major military medical treatment facilities (MTFs). However, from 2015–2019, PrEP services slowly expanded to primary care services. Defense Health Agency instruction now mandates that all MTFs have pathways in place to access PrEP services, and that PrEP remain available in both the primary care and infectious diseases subspecialty care settings across the MHS [16]. The established indications for initiation of PrEP, required laboratory testing, monitoring, and prescribing of PrEP are all conducted in accordance with the current CDC guidelines [13]. To date, no study has assessed the impact of PrEP initiation on STI incidence in the MHS. Our objective was to assess whether incidence of STIs increased among military service members who were prescribed HIV PrEP soon after it received FDA approval.

## Methods

### Patient population and data collection

Using electronic MHS pharmacy dispensation records, we identified service members without HIV infection who were prescribed the medication TDF/FTC from February 1, 2014 through June 10, 2016. These records capture prescription dispensation through retail, mail order, and MHS pharmacies worldwide which are reimbursed through the military insurance carrier, TRICARE; prescriptions paid out-of-pocket or with private insurance are not captured. Demographic, service, and sexual risk behavior characteristics of this cohort was described previously [17]. Health care providers reviewed clinical intake notes in electronic health records (EHRs) to verify initiation of PrEP and extracted reasons for initiation such as MSM contact, lack of condom use, and whether sex partners were infected with HIV [17]. Prescriptions dispensed strictly for post-exposure prophylaxis and hepatitis B treatment were excluded.

For this analysis, two health care providers independently reviewed EHRs and extracted all available positive laboratory results prior to PrEP initiation for incident *Chlamydia trachomatis* (CT), *Neisseria gonorrhea* (NG), HIV, hepatitis C virus (HCV), and syphilis. Longitudinal demographic, laboratory, and pharmacy surveillance records were obtained from the Armed Forces Health Surveillance Division’s Defense Medical Surveillance System (DMSS) from a month before PrEP initiation until March 20, 2019; electronic laboratory records were unavailable from the DMSS prior to 2010 [18].

### Case definition

A CT or NG positive laboratory result was considered a new infection if the result was at least 30 days or more after a previous positive result for the same pathogen irrespective of site of collection (for example urine/urethra, pharynx, rectum, vagina, endocervix) or after a previous negative result or, if there were no previous tests, the first positive laboratory test result. Additionally, a positive laboratory result at any site of collection for the same pathogen on the same day was considered a single infection. Any CT or NG diagnosis ≤30 days following PrEP initiation was considered to have occurred before starting PrEP. An incident STI infection was defined as any new positive laboratory result for CT and NG, syphilis, HCV, and HIV. History of syphilis before PrEP initiation was defined as the first positive rapid plasma regain (RPR) and *Treponema pallidum* antibody associated with clinical evidence of treatment. After initiation of PrEP, incident syphilis was defined as the first positive RPR test result with a titer of 1:1 or greater followed by evidence of a four-fold rise in titer and a *T*. *pallidum* positive antibody result. If an RPR titer was missing, incident syphilis was defined as a reactive RPR and a positive *Treponema pallidum* antibody following a nonreactive RPR result within the previous 12 months. Service members with no record of a positive laboratory result for STIs were assumed to be free of an STI of interest provided they received care in the MHS. Testing for STIs was determined by the treating provider, and was either based on clinical concern or recommended screening intervals after PrEP initiation based on CDC guidelines [13]. Clinical assays and tests used for testing were dependent upon availability of resources at the treating DoD facility. Generally, CT and NG were assessed using nucleic acid amplification testing (NAAT), and serologic assays were used for HIV, HCV, and syphilis (with either a non-treponemal or treponemal test).

### Data analysis

Analysis was restricted to service members with pharmacy dispensation records indicating initiation of PrEP. The frequencies of incident CT, NG, HCV, and syphilis per 100 person-years were calculated for periods on HIV PrEP and off HIV PrEP in the surveillance period. Periods off HIV PrEP included the intervals prior to HIV PrEP initiation, during medication gaps (considered greater than 14 days in duration) and after stopping HIV PrEP until the end of the surveillance period. Follow-up time prior to PrEP initiation was the interval between the later date of start of electronic health records (January 1, 2004) or start date of the first duty assignment in military service, until the date of PrEP initiation. Follow-up time on PrEP was the earliest date of TDF/FTC dispensation until the last day of pill coverage for PrEP; medication gap interval(s) greater than 14 days in duration were deducted from total pill count coverage. Follow-up time after PrEP was calculated from the day pill count ended to the earliest of either end of the study (March 20, 2019), date of separation from service or date of HIV diagnosis.

We fit Andersen-Gill (AG) recurrent event proportional hazards regression models to the data for estimation of unadjusted and adjusted hazard ratios (HRs) and 95% confidence intervals (CIs) of covariates. The counting process time interval proposed by Andersen and Gill is similar to the Cox proportional hazard regression model but allows for subjects to have non-terminal or repeated and multiple events (i.e. occurrence of recurrent or different STIs in the same patient) [19]. The primary covariates of interest in the AG models were HIV PrEP treatment, site and type of STI, and the interaction between site and type of STI and treatment. The interaction term was added to explore the relationship of STI by anatomical site on- and off PrEP, especially given screening of extragenital sites as part of PrEP services. To control for effects of other factors, covariates placed additively in the model included mediator variables at PrEP initiation: age, race/ethnicity, marital status, branch of service, pay grade, education attained, occupation, reasons for starting PrEP, and surveillance period (in years) prior to PrEP initiation. The surveillance period prior to PrEP initiation was added as a continuous variable to adjust for any effect on STI risk due to differential follow-up off PrEP compared to on PrEP. Robust variances based on sandwich estimator were computed in the AG models to adjust for correlation in repeated observations over time in a service member. Characteristics with contiguous levels having similar hazard ratios (all mediator variables except reasons for PrEP initiation) were collapsed in multivariate analyses. Multicollinearity among covariates in the multivariate model was assessed using variance inflation factor (VIF) and tolerance. Data management and statistical analyses were conducted using Statistical Analysis Software (SAS version 9.4, Cary, North Carolina, U.S) and R Studio (version 4.0.3, Boston, Massachusetts, U.S.).

### Ethical considerations

The Walter Reed Army Institute of Research Division of Human Subject Protection (#1861F, #1861G) and the U.S. Army Public Health Center (#146-12.M3) determined the project was a public health activity and not research.

## Results

A preliminary review of health records for service members who were HIV-uninfected and first prescribed TDF/FTC from February 1, 2014, through June 10, 2016 revealed that 769 service members initiated PrEP. All 10 female service members were excluded as were 4 others; one person had no evidence of pill dispensation in pharmacy records, an additional two individuals received care entirely in the civilian community, and a fourth service member, who contracted HIV in the surveillance period, was excluded due to self-reported non-initiation of PrEP treatment.

The final analysis included 755 male service members with a total 6340.4 person-years (median 7.49, interquartile range (IQR) 5.08–11.61) follow-up in the surveillance period from January 1, 2004, to March 20, 2019. Forty (5%) service members had only one visit, 116 (15%) had medication gaps (median 133.0 days, IQR 66.0–243.0 days) whereas 639 (85%) remained on PrEP continuously; only 7 (0.06%, 7/116) had gaps <30 days in duration. Service members utilized PrEP for a median 635 days (IQR 223–1089) with a median 6 (IQR 3–11) laboratory testing events and a median 77 days (IQR 30–106) between testing events; comparatively, during periods of medication gaps, service members had a median 2 (IQR 2–4) laboratory testing events and a median 83.0 days (IQR 26.0–163.0) between these events.

Overall, 477 (63%) service members had incident STIs (median 2.0, IQR 1.0–4.0), with 151 (20%) identified with only one infection and 326 (43%) having more than one STI (median 3.0, IQR 2.0–5.0); 278 (37%) had no STIs during follow-up. The overall crude STI incidence in the surveillance period was 21.4 per 100 person-years with a higher incidence on PrEP versus periods off PrEP (38.3 versus 14.4 per 100 person-years, Table 1). The most common STI while off PrEP was from NG (n = 288, 5.4 per 100 person-years), which was predominantly urogenital (4.0 per 100 person-years). *Chlamydia trachomatis* infection (n = 245, 17.4 per 100 person-years), especially of the rectum (10.1 per 100 person-years), was the most diagnosed STI while on PrEP (Table 1). One service member was diagnosed with HIV while on PrEP and 9 acquired HIV infection an average 16.3 months (range 2.7 to 24.6) after stopping PrEP.

**Table 1:** Incidence of sexually transmitted infections (STI) among 755 male service members from initiation of HIV preexposure prophylaxis, February 1, 2014—June 10, 2016, through end of follow up.

	Not on TDF/FTC^a^		On TDF/FTC^b^	
	No. of Infections	Incidence Rate per 100 person-years	No. of Infections	Incidence Rate per 100 person-years
Any STI	714	14.5	538	38.3
*N*. *gonorrhea*	288	5.8	203	14.5
Urogenital	202	4.1	65	4.6
Pharyngeal	32	0.7	68	4.8
Rectal	43	0.9	64	4.6
Other	11	0.2	6	0.4
*C*. *trachomatis*	254	5.2	245	17.5
Urogenital	164	3.3	83	5.9
Pharyngeal	7	0.1	17	1.2
Rectal	73	1.5	142	10.1
Other	10	0.20	3	0.2
*T*. *pallidum*	148	3.0	84	6.0
Hepatitis C virus	15	0.3	5	0.4
HIV	9	0.2	1	0.1

^**a**^Includes events ≤30 days after PrEP initiation, total person-time = 4936.6 person-years (gap person-time = 577.1 person-years, post-PrEP follow-up person-time = 82.9 person-years)

^**b**^Excludes infections diagnosed for test of cure and during TDF/FTC medication gaps; total person-time = 1403.9 person-years

Most service members starting PrEP were 18–28 years-old (59%, median 27.0, IQR 24.0–32.0), white (47%), and single (72%) (Table 2). The most common reasons for starting PrEP were MSM contact (89%) and/or no condom use (73%). In univariate survival analysis, service members had an 11% lower risk of incident STIs while on PrEP compared to periods off PrEP (hazard ratio (HR) 0.89, 95% CI 0.73–1.08) although it was not statistically significant (p = 0.24) (Table 2). However, after controlling for age, education level, branch of service, pay grade, occupation, reasons for starting PrEP (MSM contact, unprotected sex), and wait time prior to PrEP initiation in multivariate analysis, and the effect of PrEP on site and type of infection, risk of an STI lowered significantly by an additional 68% (adjusted HR (aHR) 0.21, 95% CI 0.11–0.40, p < .0001) compared to periods off PrEP (Table 2). Lack of condom use for starting PrEP (vs not indicated in charts: aHR 1.66, 95% CI 1.08–2.53, p = 0.006), and the wait time prior to PrEP initiation (aHR 0.70, 95% CI 0.64–0.77, p = 0.00) were independently associated with STI risk (Table 2).

**Table 2:** Characteristics of 755 male service members at initiation of HIV preexposure prophylaxis, February 1, 2014 –June 10, 2016, and unadjusted and adjusted hazard ratios of these characteristics associated with incident sexually transmitted infections (STIs) during follow up^a^.

Characteristic	N	(%)	Hazard Ratio	95% CI	P-value	Adjusted Hazard Ratio	95% CI	P-value
Age								
18–28	442	(59)	5.51	(2.54–11.99)	< .0001	1.03	(0.66–1.62)	1.00
29–40	257	(34)	1.51	(0.69–3.29)	0.31	Referent	-	-
41–48	43	(6)	1.42	(0.60–3.38)	0.43			
49+	13	(2)	Referent	-	-			
Race/ethnicity								
White	356	(47)	Referent	-	-	Referent	-	-
Black	144	(19)	1.16	(0.86–1.55)	0.33	1.26	(0.83–1.92)	0.92
Hispanic	146	(19)	1.26	(0.95–1.66)	0.11	1.36	(0.93–1.99)	0.27
Asian	37	(5)	1.62	(1.00–2.63)	0.05	1.07	0.63–1.81)	1.00
American Indian	9	(1)	0.43	(0.19–0.96)	0.04			
Other	50	(7)	1.11	(0.69–1.79)	0.66			
Unknown	13	(2)	0.65	(0.28–1.48)	0.30			
Marital status								
Single, never married	545	(72)	1.32	(1.02–1.70)	0.03	1.01	(0.70–1.45)	1.00
Married	179	(24)	Referent	-	-	Referent	-	-
Other	31	(4)	0.96	(0.54–1.69)	0.88			
Branch of service								
Navy	359	(48)	1.39	(1.05–1.83)	0.02	1.01	(0.66–1.56)	1.00
Marine Corps	40	(5)	1.77	(1.05–2.98)	0.03			
Army	202	(27)	1.24	(0.90–1.71)	0.18	1.13	(0.69–1.85)	1.00
Air Force	154	(20)	Referent	-	-	Referent	-	-
Pay grade								
E1-E4	249	(33)	4.08	(3.16–5.26)	< .0001	1.27	(0.83–1.95)	0.91
E5-E9	168	(22)	Referent	-	-	Referent	-	-
Officer	338	(45)	0.79	(0.59–1.08)	0.14			
Highest level of education attained								
High School or less	443	(59)	2.34	(1.59–3.45)	< .0001	1.11	(0.76–1.62)	1.00
Some College	82	(11)	1.47	(0.92–2.35)	0.11	Referent	-	-
Bachelor’s Degree	117	(16)	1.45	(0.91–2.33)	0.12			
Higher than Bachelor’s degree	79	(10)	1.00	-	-			
Unknown	34	(5)	1.41	(0.74–2.67)	0.29			
Occupation								
Communication/intelligence	214	(28)	Referent	-	-	Referent	-	-
Healthcare	189	(25)	0.84	(0.63–1.11)	0.21	0.93	(0.61–1.42)	1.00
Repair/engineering	170	(23)	1.04	(0.78–1.39)	0.80	1.03	(0.68–1.58)	1.00
Motor transport	14	(2)	2.02	(0.9–4.56)	0.09			
Other	118	(16)	0.78	(0.56–1.08)	0.13	0.88	(0.58–1.33)	1.00
Infantry/artillery/combat engineering	39	(5)	0.71	(0.43–1.19)	0.19			
Pilot/aircrew	11	(1)	0.39	(0.15–1.00)	0.05			
Reasons for initiating PrEP^b^								
Male-to-male sexual contact	675	(89)	2.91	(1.91–4.41)	< .0001	1.20	(0.63–2.28)	1.00
No condom	550	(73)	2.86	(2.21–3.69)	< .0001	1.66	(1.08–2.53)	0.006
HIV+ sexual partner	230	(30)	0.74	(0.59–0.93)	0.01	1.00	(0.71–1.42)	1.00
Surveillance period (in years) prior to PrEP initiation; median (IQR)	4.9	(2.59–9.40)	0.73	(0.69–0.76)	< .0001	0.70	(0.64–0.77)	0.00
On emtricitabine (TDF)/tenofovir (FTC) vs off	-	-	0.89	(0.73–1.08)	0.24	0.21	(0.11–0.4)	< .0001
Type and site of infection								
Urogenital *N*. *gonorrhea*	220	(29)	Referent	-	-	Referent	-	-
Pharyngeal *N*. *gonorrhea*	34	(5)	2.41	(1.55–3.74)	< .0001	1.03	(0.49–2.2)	1.00
Rectal *N*. *gonorrhea*	25	(3)	4.08	(2.55–6.52)	< .0001	1.92	(0.97–3.8)	0.08
Other *N*. *gonorrhea*	8	(1)	2.43	(1.07–5.53)	0.03	3.24	(0.74–14.17)	0.31
Urogenital CT	124	(16)	1.99	(1.55–2.55)	< .0001	1.79	(1.13–2.82)	0.00
Pharyngeal CT	8	(1)	4.73	(2.36–9.48)	< .0001	1.25	(0.31–4.99)	1.00
Rectal CT	67	(9)	3.58	(2.58–4.98)	< .0001	1.36	(0.8–2.31)	0.85
Other CT	8	(1)	1.89	(0.69–5.19)	0.21	2.84	(0.62–12.86)	0.59
Syphilis	112	(15)	1.58	(1.23–2.02)	0	1.21	(0.73–1.99)	1.00
HCV	16	(2)	0.9	(0.35–2.31)	0.83	0.75	(0.07–7.63)	1.00
HIV	133	(18)	0.04	(0.02–0.08)	< .0001	0.02	(0.01–0.07)	0.00
Interaction of site and type of infection and TDF/FTC								
Urogenital *N*. gonorrhea*TDF/FTC	-	-	-	-	-	Referent	-	-
Pharyngeal *N*. gonorrhea*TDF/FTC	-	-	-	-	-	8.62	(2.82–26.34)	< .0001
Rectal *N*. gonorrhea*TDF/FTC	-	-	-	-	-	5.74	(2.1–15.73)	< .0001
Other *N*. gonorrhea*TDF/FTC	-	-	-	-	-	0.61	(0.05–7.95)	1.00
Urogenital CT*TDF/FTC	-	-	-	-	-	2.99	(1.26–7.12)	0.002
Pharyngeal CT*TDF/FTC	-	-	-	-	-	10.75	(1.61–71.9)	0.002
Rectal CT*TDF/FTC	-	-	-	-	-	6.38	(2.7–15.07)	< .0001
Other CT*TDF/FTC	-	-	-	-	-	1.82	(0.08–41.66)	1.00
Syphilis*TDF/FTC	-	-	-	-	-	3.66	(1.56–8.56)	< .0001
HCV*TDF/FTC	-	-	-	-	-	2.02	(0.08–48.15)	1.00
HIV*TDF/FTC	-	-	-	-	-	1.87	(0.06–58.39)	1.00

^a^ Andersen-Gill (AG) recurrent event proportional hazards regression models were fit to the data for estimation of an incident sexually transmitted infection (STI). Primary covariates considered in unadjusted and adjusted AG models were HIV pre-exposure prophylaxis (PrEP) treatment, site and type of STIs, and the interaction between site and type of STI and treatment (adjusted only); other explanatory or mediator variables were age, race/ethnicity, marital status, branch of service, pay grade, education attained, occupation, reasons for starting PrEP, and the surveillance period (in years) prior to PrEP initiation.

^b^ Absence of documentation in clinical records for each of the reasons for starting PrEP (MSM contact, lack of condom use, HIV-infected sex partner) was considered the referent category.

In stratifying the risk of STI by site and type of infection and PrEP use, the risk of diagnosis of urogenital *N*. *gonorrhea* was significantly lower during PrEP (aHR 0.21, 95% CI 0.12–0.38, p < .0001) in contrast to the risk of STI to periods off PrEP (Table 3). In contrast, the risk of diagnosis of extragenital NG infection (pharyngeal NG: aHR 1.84, 95% CI 0.82–4.13, p = 0.30; rectal NG: aHR 1.23, 95% CI 0.60–2.51, p = 1.00) and extragenital CT infection (pharyngeal CT: aHR 2.30, 95% CI 0.46–11.46, p = 0.81; rectal CT: aHR 1.36, 95% CI 0.81–2.31, p = 0.66) was greater on PrEP compared to off PrEP although it did not reach statistical significance (Table 3).

**Table 3:** Adjusted hazard ratio (HR) of incident sexually transmitted infections (STIs) by site and type of STI and use of HIV pre-exposure prophylaxis (PrEP) medication (TDF/FTC).

	Off PrEP (TDF/FTC = 0)			On PrEP (TDF/FTC = 1)			On PrEP Contrasted to Off PrEP (TDF/FTC = 1–0) ^b^		
Site and type of infection	Adjusted ^a^ HR (95% CI)		p-value	Adjusted ^a^ HR (95% CI)		p-value	Adjusted ^a^ HR (95% CI)		p-value
Urogenital *N*. *gonorrhea*	Referent			0.21	(0.12–0.39)	< .0001	0.21	(0.12–0.38)	< .0001
Pharyngeal *N*. *gonorrhea*	1.03	(0.51–2.11)	1.00	1.90	(0.95–3.8)	0.09	1.84	(0.82–4.13)	0.30
Rectal *N*. *gonorrhea*	1.92	(1.01–3.66)	0.05	2.36	(1.27–4.38)	0.00	1.23	(0.60–2.51)	1.00
Other *N*. *gonorrhea*	3.24	(0.8–13.12)	0.19	0.42	(0.06–2.82)	0.96	0.13	(0.01–1.24)	0.11
Urogenital *C*. *trachomatis*	1.79	(1.16–2.75)	0.00	1.14	(0.62–2.09)	1.00	0.64	(0.37–1.12)	0.23
Pharyngeal *C*. *trachomatis*	1.25	(0.34–4.64)	1.00	2.87	(0.89–9.23)	0.12	2.30	(0.46–11.46)	0.81
Rectal *C*. *trachomatis*	1.36	(0.83–2.24)	0.68	1.86	(1.1–3.14)	0.007	1.36	(0.81–2.31)	0.66
Other *C*. *trachomatis*	2.84	(0.68–11.89)	0.41	1.10	(0.08–14.55)	1.00	0.39	(0.02–6.11)	0.99
Syphilis	1.21	(0.75–1.94)	0.99	0.94	(0.55–1.62)	1.00	0.78	(0.46–1.32)	0.89
Hepatitis C virus	0.75	(0.08–6.77)	1.00	0.32	(0.04–2.77)	0.88	0.43	(0.03–7.23)	1.00
HIV	0.02	(0.01–0.07)	0.00	0.01	(0–0.2)	< .0001	0.40	(0.02–7.99)	1.00

^a^ Adjusted for characteristics at PrEP initiation age, race, branch of service, pay grade, education attained, marital status, occupation, reasons for initiating PrEP (MSM contact, lack of condom use, HIV-infected sex partner), and surveillance period (in years) prior to PrEP initiation.

^b^ Adjusted HR for TDF/FTC = 1 was divided by the adjusted HR for TDF/FTC = 0. The p-values in the TDF/FTC (1–0) column test the null hypothesis that (HR1/HR0) = 1 (i.e., no difference in HRs) for all site and types of infection.

## Discussion

After adjustment for the effect of PrEP on site and type of infection, socio-demographic characteristics and reasons for starting PrEP, overall STI risk decreased significantly while on PrEP among male service members who initiated PrEP in the MHS from February 1, 2014 to June 10, 2016. However, stratification of risk by site and type of infection and treatment indicated the risk of diagnosis of extragenital bacterial STIs was much greater while on PrEP vs off PrEP although statistical significance was not reached. Lack of condom use as the reason for starting PrEP and wait timeprior to PrEP initiation were independently associated with STI risk.

STI screening is likely to occur more frequently in the setting of PrEP adherence and follow-up care when following CDC PrEP guidelines, than off PrEP thus allowing for more opportunities to diagnose asymptomatic infections. Additionally, testing or screening of extragenital sites is more likely to occur, leading to higher rates of diagnosis of pharyngeal/rectal CT and/or NG. Moreover, in this analysis, more than half of service members were cared for by dedicated sub-specialists with experience in HIV and STI care, and thus those engaged in PrEP services also receive more STI prevention counseling, potentially contributing to decreases in STI risk while on PrEP [17]. These data also suggest service members who were at high risk of STIs appropriately sought PrEP care and were relatively early adopters of prophylactic treatment. It is also possible the observed effect may be an artifact of differential follow-up on versus off PrEP. Although our analysis controlled for overall longer wait time prior to PrEP initiation, it was not possible to control for asymptomatic extragenital CT or NG infections that may have occurred in periods off PrEP for which service members did not seek treatment in the military health system. Other studies have suggested that some level of immunity may have accrued over time from these infections offering reduced susceptibility to reinfection which may have contributed to an overall reduced risk of STI artifactually associated with PrEP use [20–22].

Evidence to date for increased STI acquisition among PrEP users has been mixed. STI positivity did not differ between treatment and control groups nor was an increase in STIs observed in the PrEP group in efficacy trials conducted before July 5, 2020 [23]. These findings may have been confounded by study design and inclusion criteria since patients enrolled in trials were at high risk of HIV infection and blinding to PrEP allocation and whether treatment was received may have influenced sexual behavior. However, an increased odds of overall STI diagnosis was observed (odds ratio (OR), 1.24, 95% CI, .99–1.54) in a meta-analyses of open-label PrEP studies (2014–2017), although it was not statistically significant despite significantly increased odds of rectal STIs (OR, 1.39; 95% CI, 1.03–1.87), especially for chlamydia (OR 1.59, 95% CI 1.19–2.13) [24]. Although these studies were limited by lack of pre-enrollment analysis of STI positivity since studies compared STI positivity at baseline to follow-up. In studies which did address STI history before PrEP was initiated, an increase in incidence (Nguyen et al. adjusted rate ratio (aRR) 1.39, 95% CI 0.98–1.96; Beymer et al. RR 1.36, 95% CI 1.06–1.74; Traeger et al. aRR 1.21, 95% CI 1.06–1.39) was observed although statistical significance was not reached in all analyses [12, 25, 26]. These cohort studies compared STI diagnoses among MSM in sexual health care in up to 12 months preceding PrEP initiation to follow-up. While our analysis demonstrated an increase in crude incidence during periods on PrEP versus off PrEP, we were able to show that overall STI risk decreased, contrasting with trends from other cohort studies. The analytic approach used here considered interaction and stratified STI by anatomic site and PrEP treatment. This elucidated that overall increases in STI incidence on PrEP were due to the frequency of screening of extragenital sites where a higher proportion of infections are asymptomatic. A finding which was further supported by lowered risk on PrEP of urogenital CT/NG which are primarily symptomatic. While the 2010 CDC STI guidelines recommended extragenital screening MSM, potentially at 3–6 months for some individuals, this was incumbent on the patients self-identifying and presenting to care; contrasting with a more compulsory follow-up engagement for those on PrEP due to the intervals for HIV-testing and TDF/FTC renewal [13, 27]. This further highlights the non-uniform impact that PrEP initiation has on non-HIV STIs.

In our analysis, despite the independent association between STI risk and lack of condom use at baseline, STI incidence (38.3 per 100 person-years) was much lower compared to reports of similar cohort analyses (83.5–107.7 per 100 person-years) [12, 25, 26]. It is possible the difference may be due to shorter follow-up time in these studies and/or differences in STI prevalence across geographically different sexual networks since level of condom use was not associated with STI risk in one of the studies [12].

Our analysis has several limitations. Differential screening for STIs while on PrEP to periods off PrEP may have introduced surveillance bias and differential detection of STIs during follow-up. However, a strength of this analysis was that surveillance for any prior STI diagnosis from entry into service to initiation of PrEP care served as a control period and an opportunity for adjustment for the length of the time period; this may have minimized large differences compared to studies with surveillance that was limited to only 3 to 12 months before PrEP initiation. Second, since pharmacy dispensation records were used to assess PrEP coverage and not self-reported pill counts or TDF/FTC blood levels at each visit, follow-up time in PrEP care could have been misclassified leading to error in estimations of risk. Third, the case definition of syphilis changed from before PrEP initiation to follow-up. This may have led to error in estimation of syphilis risk and, consequently, STI risk. Lastly, the cohort of PrEP users we followed, and findings associated with this group, are likely not representative of current uptake of PrEP or clinicians’ prescribing patterns. This cohort was largely cared for by sub-specialists with advanced training in HIV and STI-related care due to the relative novelty of PrEP in this population during the study period. However, describing this early cohort of PrEP users’ STI risk provides a frame of reference for comparative trend analysis, especially since the scope of PrEP services in the military was expanded to include primary care in 2019 [16].

In conclusion, although uptake of PrEP may have led to a modest increase in diagnosis of STIs, especially of extragenital infections, the data suggest entry into PrEP care was associated with an overall reduced risk of STI. This is likely multifactorial: service members who sought PrEP may have recognized their existing high risk of HIV acquisition and adopted PrEP early and they may have benefited from greater STI prevention counseling. Then again, the observed reduction in overall risk of STI may have been an artifact of the longer surveillance period prior to initiation of PrEP where repeat asymptomatic undiagnosed infection may have reduced susceptibility to CT and NG infections. Nonetheless, the data suggest that routine STI screening should remain a cornerstone of PrEP care given the burden that increased STI rates have on public health, as well as the role that frequent follow-up and counseling can play in mitigating incident STIs. Long term studies on STI incidence in the PrEP population could help inform policy and clinical guideline recommendations to mitigate risk.
